# Luminescent Properties of Eu^3+^-Doped Hybrid SiO_2_-PMMA Material for Photonic Applications

**DOI:** 10.3390/mi9090441

**Published:** 2018-09-01

**Authors:** Pablo Marco Trejo-García, Rodolfo Palomino-Merino, Juan De la Cruz, José Eduardo Espinosa, Raúl Aceves, Eduardo Moreno-Barbosa, Oscar Portillo Moreno

**Affiliations:** 1 Facultad de Ciencias Físico Matemáticas, Benemérita Universidad Autónoma de Puebla, Avenida San Claudio y 18 Sur, Colonia San Manuel, Ciudad Universitaria, C.P. 72570 Puebla, Mexico; pablo.trejogarcia@alumno.buap.mx (P.M.T.-G.); 217570100@alumnos.fcfm.buap.mx (J.D.l.C.); espinosa@fcfm.buap.mx (J.E.E.); emoreno@fcfm.buap.mx (E.M.-B.); 2 Departamento de Investigación en Física, Universidad de Sonora, Apartado Postal 5-088, C.P. 83190 Hermosillo, Sonora, Mexico; raceves@cifus.uson.mx; 3 Facultad de Ciencias Químicas, Benemérita Universidad Autónoma de Puebla, P.O. Box 1067, Colonia San Manuel, Ciudad Universitaria, C.P. 72570 Puebla, Mexico; osporti@yahoo.com.mx

**Keywords:** europium, luminescence, hybrid materials, microdevices

## Abstract

Hybrid organic-inorganic materials are of great interest for various applications. Here, we report on the synthesis and optical characterization of silica-PMMA samples with different Eu^3+^ molar concentrations. The optical properties of this material make it suitable for photonic applications. The samples were prepared using the sol-gel method, mixing tetraethyl orthosilicate (TEOS) as a silica glass precursor and methyl methacrylate (PMMA) as a polymer component. Europium nitrate pentahydrate was then added in six different molar concentrations (0.0, 0.1, 0.25, 0.5, 0.75, and 1%) to obtain as many different samples of the material. The absorption spectra were obtained applying the Kubelka–Munk formula to the diffuse reflectance spectra of the samples, all in the wavelength range between 240 and 2500 nm. The emission and excitation measurements were made in the visible range. Five bands could be identified in the emission spectra, related to electronic transitions of the ion Eu^3+^ (^4^D_0_→^7^F*_i_*, *i* from 0 to 4). In the excitation spectra, the following bands were detected: ^7^F_0_→^5^G_3_ (379 nm), ^7^F_0_→^5^G_2_ (380 nm), ^7^F_0_→^5^L_6_ (392 nm), ^7^F_0_→^5^D_3_ (407 nm), ^7^F_0_→^5^D_2_ (462 nm), and ^7^F_0_→^5^D_1_ (530 nm). The emission decay times were measured for the different samples and showed an inverse dependence with the Eu^3+^ concentration.

## 1. Introduction

The study and development of hybrid materials has taken off since the end of 20th century and the beginning of the 21st. This is in spite of the fact that hybrid materials were developed for thousands of years when the production of paints was the driving force to try novel mixtures of dyes and/or inorganic pigments. This takeoff has happened due to the availability of novel physico-chemical characterization methods, as well as new perspectives of material creation made possible by nanoscience. Since then, bottom-up strategies going from the molecular level up to material design have led to the creation of materials with very different and applicable physico-chemical properties. Despite all these changes, when the synthetization of hybrid materials is considered, the sol-gel method has continued to be widely used, as it is cost effective and allows for the synthesis of materials of high purity and homogeneity. On the other hand, the search for a better and better matrix able to host a dopant has been a key objective of research for years. An example of this are SiO_2_ matrixes, which incorporate lanthanides, are homogenous and transparent, and have controlled porosity; they can be useful in many photonic applications, including the development of doped fiber amplifiers (DFA) for continuous and pulsed lasers, single-mode silica optical fibers that have the ability to provide high bandwidth and long distance communications, or, finally, materials for thermoluminescent applications [1,2].

Methyl methacrylate (PMMA) is an organic polymer, which is transparent, malleable and flexible. This material has recently been employed to make polymeric optical fibers (POFs) [3], as well as highly tunable Bragg gratings [4]. Kuriki and Koike [5] synthesized lanthanide (La)-doped POFs and showed that the fibers were capable of incorporating the inorganic host in high concentrations. They also found that the lanthanides in these systems were pumped more effectively than in conventional crystal systems. This happened because of energy transfer processes in the chelate complexes [5]. Basu and Vasantharajan doped polystyrene, polymethylmethacrylate, and polyurethane matrices with europium for the fabrication of temperature-sensitive coatings (TSP) [6]. The undoped hybrid PMMA-SiO_2_ presents other characteristics that its components do not present; for example, it has a higher glass transition temperature, a higher optical transparency, a better thermal stability [7], a better adhesion strength than the pure PMMA that allows it to be employed as anticorrosive coating [8], and size-controlled silica particles, which allow it to effectively reduce the gas permeability of the polymer membrane [9].

On the other hand, the Eu^3+^ ion has played an important role in the development of many optical devices, such as lasers, phosphor materials, coatings, luminescent probes, POFs, displays, and so on. This great interest is based on the intense luminescence it emits in the red range, which is generated by the large energy gap between the ground and the first excited state of this element. Moreover, Eu^3+^ is widely used because the ground energy state (^7^F_0_) and the most important emitting excited state (^5^D_0_) are nondegenerate and do not split because of the crystal-field effect. This allows us to easily understand the absorption and emission spectra and their dependence on the environment in which the Eu^3+^ is located. Based on this fact, Eu^3+^ has been widely used as a luminescent probe for host structures and defect studies [10]. The most important transitions in the luminescence spectra of Eu^3+^ are those from the ^5^D_0_ excited state to ^7^F*_J_* levels, (with a low *J* value equal to 0, 1, or 2). The interpretation of the spectra turns out to be easy because of the small number of possible crystal-field transitions. Other interesting characteristics refer to the facts that, because the different ^5^D_0_→^7^F*_J_* lines are well separated, overlapping between the crystal-field levels is difficult [11] and that the luminescence intensity ratio of the magnetic (^5^D_0_→^7^F_1_) and electric bands (^5^D_0_→^7^F_2_), both in the red range of the spectrum, shows a larger symmetry factor with a larger symmetry of local crystal fields [12].

In this study, six hybrid samples, made of inorganic silica and organic PMMA, were synthetized using the sol-gel method. Five samples were doped with different molar concentrations of Eu^3+^, and one was left undoped. The goal of the study was to characterize the optical properties of these materials because of their potential applications. Due to their transparency, the ease of their fastening to silica and to substrates—because of their vitreous part, increased flexibility conferred by the polymer component, increased mechanical properties, and the fluorescence properties conferred by the dopant—and their capacity to change the refractive index by changing the ratio between the precursors, these materials can be used in the near future as a coating for optical fibers for the development of optical devices, such as sensors, which can act under the excitation of the dopant as a coating layer for scratch resistance or corrosion protection and as an optical filter device. In addition, because the material is made through the sol-gel method, the manufacturing of fibers at room temperature is allowed.

## 2. Materials and Methods 

*Material Preparation*: Six different SiO_2_-PMMA samples were prepared with the sol-gel method. During this process, tetraethyl orthosilicate (TEOS), PMMA, and ethanol were mixed. After that, trimethoxysilyl propyl methacrylate (TMSPM) was added and used as a bonding agent between the polymer and the SiO_2_ molecules. The molar ratios were 1:1:0.22:4.75:4.75 (TEOS/PMMA/TMSPM/H_2_O/Ethanol). Sodium hydroxide (NaOH) was added as a catalyst for the hydrolysis to increase the pH of the solution up to a value of 9. Benzoyl peroxide (BPO) was used as a catalyst for the methyl methacrylate (MMA) polymerization in a 1 mass % ratio with respect to the amount of PMMA used [3]. After these steps were completed, europium nitrate pentahydrate was added in molar concentrations of 0, 0.1, 0.25, 0.5, 0.75, and 1 mol %. All this to form one undoped sample and five Eu-doped samples of the hybrid material studied in this work. The gel formation process lasted for a period of 25 days, finishing when the samples were completely dry. 

*Spectra Characterization*: Absorption spectra were obtained applying the Kubelka–Munk equation as follows:

(1 − *R*_∞_)^2^/2 × *R*_∞_ ≡ *F*(*R*) (1)

where *F*(*R*) is the Kubelka–Munk function and *R_∞_* is the reflectance of a layer so thick as to completely hide the substrate. For this investigation, the diffuse reflectance spectra of the samples were recorded in the range between 240 and 2500 nm using a CARY 5000 spectrophotometer (Agilent Technologies, Santa Clara, CA, USA). The band gap energy (*Eg*) was calculated by the interpolation of a line in the graph of (*F*(*R*)*hν*)*^n^* versus *hν* (Tauc plot); “*n*” takes the values of 0.5 or 2 (depending on whether the allowed transition is direct or indirect, respectively) and *hν* is the corresponding photon energy [13]. Emission and excitation sample spectra were always recorded at room temperature (RT). For the emission spectra, the setup array was in frontal face mode in a Nanolog Spectrofluorometer (Jobin-Yvon Horiba, Horiba, Ltd., Kyoto, Japan) equipped with double grating in both the excitation and emission monochromators and with a Xenon lamp of 450 W. For the emission spectra, a range of wavelengths greater than the ones used for excitation were used. 

Decay time calculations: Because the decay time of Eu^3+^ is in the order of milliseconds, the measurements of the fluorescent decay time were carried out at RT, using a time-correlated single photon-counting (TCSPC) Fluorolog3-TCSPC (Jobin-Yvon Horiba, Horiba, Ltd.). This was a hybrid steady-state (continuous wave (CW)) system with time-correlated fluorescence dynamics. It was equipped with double grating in both the excitation and emission monochromators, a 450 W Xenon lamp for CW measurements, and a Xenon pulsed lamp (3 ms pulse duration). The excitation wavelength used to carry out the experiments was 393 nm, which corresponded to the stronger wavelength that could excite the electronic state ^5^D_0_ (614 nm). Finally, a natural logarithm function was applied to the spectra, and a linear fit was calculated; the slope of this line gave us the fluorescence decay time of each sample.

## 3. Results

**Figure 1:**
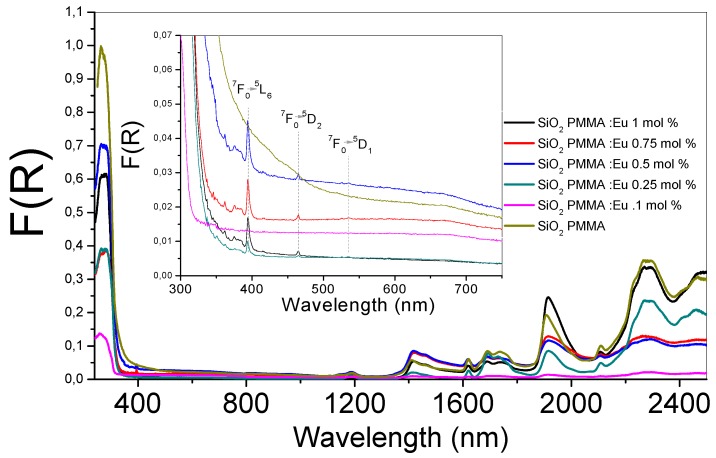
Absorption spectra of the six SiO_2_- methyl methacrylate (PMMA) samples. The absorption spectra for the undoped and doped hybrid samples are shown in the wavelength range between 240 and 2500 nm. In the inset, a zoom of the range 300–750 nm is presented, where the electronic transitions of the Eu^3+^ ion are identified. The line color of each sample is the same in both graphs.

In Figure 1, the absorption spectra of the six SiO_2_-PMMA samples under investigation are presented. The wavelengths varied in the 240–2500 nm range. In the inset image of Figure 1, an enlargement of the wavelength region between 300 and 750 nm is presented. It can be observed that the absorption edge for all the samples was approximately 350 nm. All the samples presented the same bands with different amplitudes. In the inset image, due to the Eu^3+^ ion presence, three bands were identified that were associated with the electronic transitions ^7^F_0_→^5^L_6_ (392 nm), ^7^F_0_→^5^D_2_ (460 nm), and ^7^F_0_→^5^D_1_ (538 nm). The first band represented the most intense absorption and was observable for all the doped samples. It appeared due to the direct excitation into the 4f^6^ levels of the Eu^3+^ ions [11]. The band associated with ^7^F_0_→^5^D_2_ was an electric dipole transition and was used to determine the position of the ^5^D_2_ level; it was observable for dopant concentrations higher than 0.1 mol %. The magnetic dipole transition (^7^F_0_→^5^D_1_) presented small intensities and was observed only for the samples doped at 0.75 and 1 mol %.

**Figure 2:**
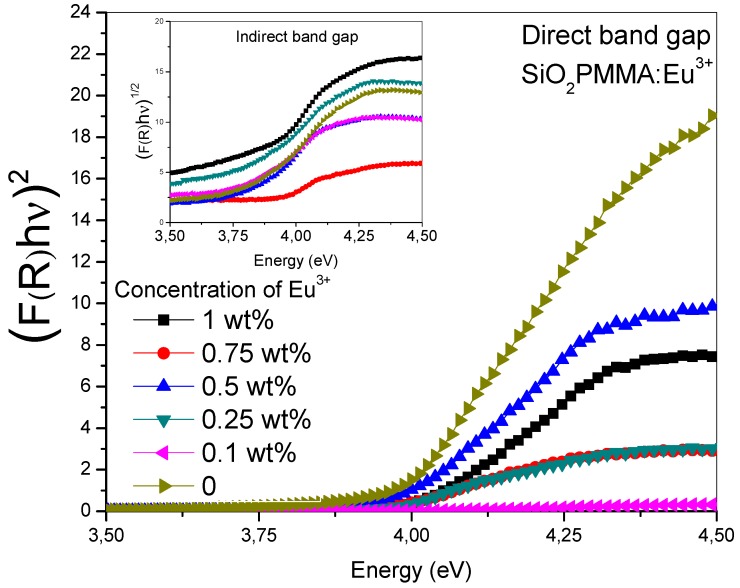
(*F*(*R*)*hν*)*^n^* vs photon energy (Tauc plot) in the absorption edge range for the SiO_2_-PMMA:Eu^3+^ samples. In the inset, the (*F*(*R*)*hν*)*^1/2^* vs photon energy relation is plotted for *n* = 0.5, while in the main graph *n* = 2.

Figure 2 was obtained drawing the Tauc plot [(*F*(*R*)*hν*)*^n^* vs hν] for the absorption spectra. The main graph corresponds to an *n* value of 2 and the inset to *n* = 0.5. It can be observed that for *n* = 2 in the absorption edge (3.75−4.25 eV), the samples presented a linear behavior, while in contrast with *n* = 0.5, they presented a polynomial behavior. This property is typical of a direct band gap material. The results reported in the Table 1 show the direct gap value for each dopant concentration; the data were calculated from the extrapolated crossing of the linear segment of the absorption edge with the energy-axis. These values showed that there were no significant changes in the band gap due to dopant presence.

**Figure 3:**
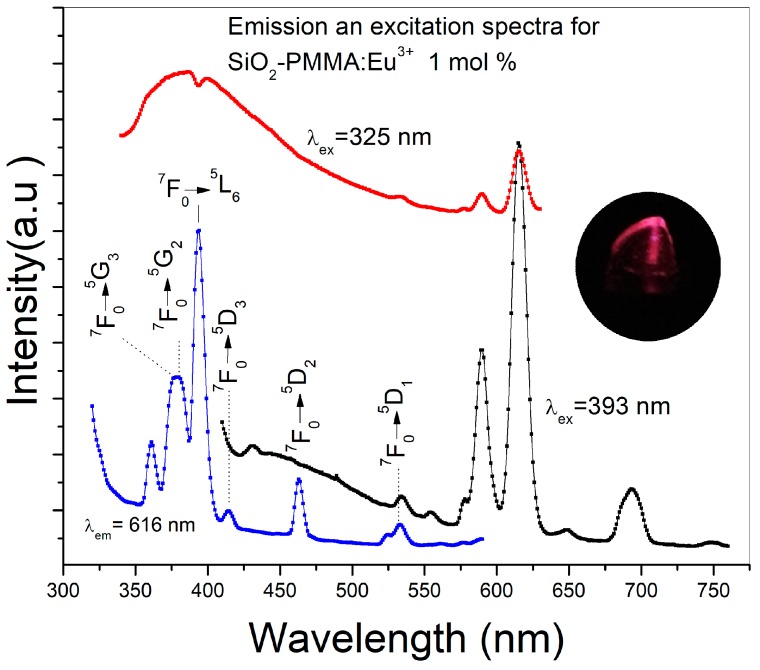
Emission and excitation spectra of a SiO_2_-PMMA:Eu^3+^ sample doped at 1 mol % using different excitation wavelengths. The red line corresponds to the excitation at 325 nm and the black line to the excitation at 393 nm. The excitation spectrum was obtained by detection at 616 nm (blue line). Electronic transitions associated with higher energies were identified in the excitation spectrum. In the inset, an image of the emission of the sample using an excitation wavelength of 393 nm can be observed.

Figure 3 presents the emission spectra using 325 and 393 nm as excitation wavelengths and the excitation spectrum detected at 616 nm of a SiO_2_-PMMA:Eu^3+^ sample with 1 mol % Eu^3+^. The excitation wavelength of 325 nm was used to observe the complete emission band due to the SiO_2_-PMMA matrix, which is in the range of 340–500 nm. In contrast, the excitation wavelength of 393 nm was used to excite the Eu^3+^ ions and to induce photoluminescence due to the direct population of the 4f levels. In the same figure, the excitation spectrum recorded at 616 nm displays a set of bands associated with the 4f electronic transitions: ^7^F_0_→^5^G_3_ (377 nm), ^7^F_0_→^5^G_2_ (380 nm), ^7^F_0_→^5^L_6_ (392 nm), ^7^F_0_→^5^D_3_ (413 nm), ^7^F_0_→^5^D_2_ (462 nm), and ^7^F_0_→^5^D_1_ (530nm) [11,14]. Because the emission spectrum of the SiO_2_-PMMA matrix (340−500 nm) presents a decrement at around 392 nm and the excitation spectrum at 616 nm has the most intense band associated with the electronic transition ^7^F_0_→^5^L_6_ (392 nm) in the same wavelength position, it can be argued that the decrement is caused by the radiative energy transfer from the matrix to the Eu^3+^. 

**Figure 4:**
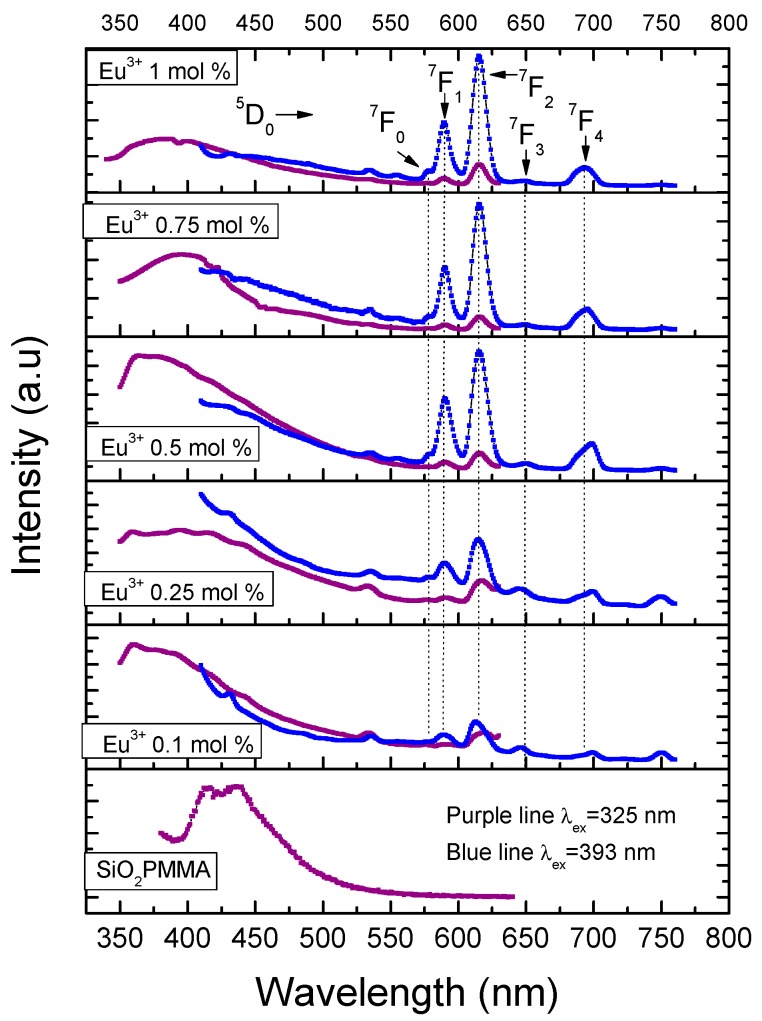
Emission spectra after excitation at 325 nm (purple line) and 393 nm (blue line) for the different hybrid material samples. The intensities of the two emission spectra are not at the same scale; they have been normalized to fit into the same plot frame.

Figure 4 presents the emission spectra for all the samples using excitation wavelengths of 325 and 393 nm. The excitation wavelength of 325 nm was used to observe the matrix luminescence, which is composed by a broad band from 350 nm to 430 nm. The optimum excitation wavelength of 393 nm was used to observe the electronic transitions of Eu^3+^. For this ion, five bands related to the electronic transitions ^5^D_0_→^7^F*_i_* (*i* from 0 to 4) with maxima in 578, 590, 615, 649, and 693 nm could be identified. It should be noted that for the dopant concentrations used, fluorescence quenching was never observed; also, due to the fact that the dopant concentrations used were small, radiative energy transfer was not observed for the samples with dopant concentration smaller than 1 mol %.

**Figure 5:**
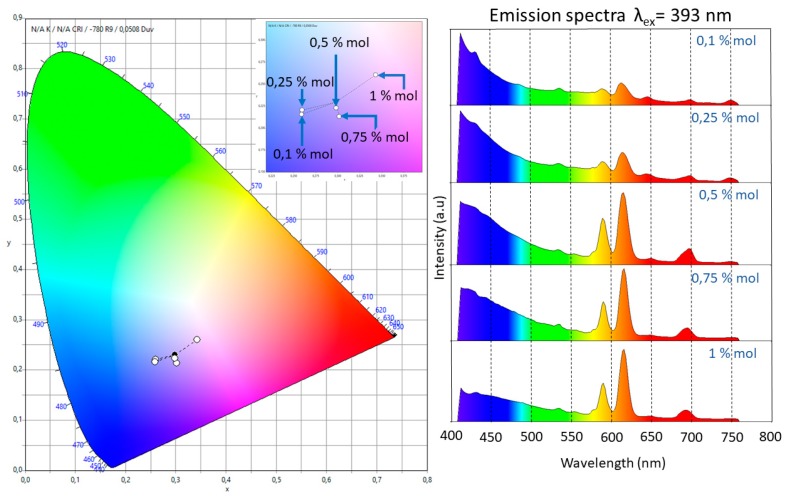
CIE 1931 2º chromaticity diagram. Color chromaticity of the emission in the range of 410–760 nm of the five doped samples under the excitation wavelength at 393 nm. The inset in the diagram represents an amplification of the blue-pale red zone. The red shift in the emission was observed as an increase of the molar relation of Eu^3+^.

The presence of a single band associated with the ^5^D_0_→^7^F_0_ transition, which could not be split by the crystal field, indicated that the Eu^3+^ ion occupied a single site with C_nv_, C_n_, or C_s_ symmetries [11]. The intensity of the ^5^D_0_→^7^F_1_ transition, which is related to the magnetic dipole nature, grew as did the molar concentration of Eu^3+^. Even though this electronic transition is observed in all the doped samples, for the slightly doped samples (0.1 and 0.25 mol %) a blue-pink emission was observed due to the fact that the emission of the matrix has a strong component in the blue region. The ^5^D_0_→^7^F_2_ band has an electric dipole nature, and the increase on the intensity of this band, which was associated with low symmetries of the Eu^3+^, grew faster than that of the ^5^D_0_→^7^F_1_. This fact could be confirmed trough the measurement of the ratio *I*(^5^D_0_→^7^F_2_)*/I*(^5^D_0_→^7^F_1_). The following values were obtained: 1.185 (0.1 mol %), 1.212 (0.25 mol %), 1.574 (0.5 mol %), 1.907 (0.75 mol %), and 1.957 (1 mol %). These results meant that the samples tended to have a lower symmetry as the dopant increased and confirmed the red shift of the luminescence observed in the samples due to the fast growth in intensity of this band compared with the others. This fact is also observable in the International Commission on Illumination (CIE) chromaticity diagram shown in Figure 5. In this diagram, the shift from the blue region to the pale red region can be observed as the dopant concentration increases.

The red shift behaviour had been previously reported and observed in (Y_1-X_Eu_x_)_2_O_2_S, (Y_1-X_Eu_x_)_2_O_3_, and (Y_1-X_Eu_x_)_2_VO_4_ materials and was associated with the favoring of the ^5^D_0_ level [15]. The transition ^5^D_0_→^7^F_3_ was very weak for all the molar concentrations of the dopants. The intensity ratio between this band and that associated with the ^5^D_0_→^7^F_1_ band decreased as the molar concentration grew.

The observation of a weak emission associated with the ^5^D_0_→^7^F_4_ transition for all the samples confirmed the low symmetry of the material and supported the idea that the chemical composition of the host matrix influenced the intensity of this band. In other words, when SiO_2_ is present in the matrix, the ^5^D_0_→^7^F_2_ transition is the most important in the emission spectrum, while when SiO_2_ is absent, the main role is played by the ^5^D_0_→^7^F_4_ transition. This was first observed by Bortoluzzi et al. [16] for Eu(Tp)_3_ in PMMA polymer matrix and Eu(Tp)_3_ (Tp = hydrotris (pyrazol-1-yl) borate). A similar behavior was observed by Blasse and Bril for GdOCl:Eu^3+^, in which the transition ^5^D_0_→^7^F_4_ dominated the spectrum, while for GdOBr:Eu^3+^ it was the ^5^D_0_→^7^F_2_ transition that was dominant [17].

**Figure 6:**
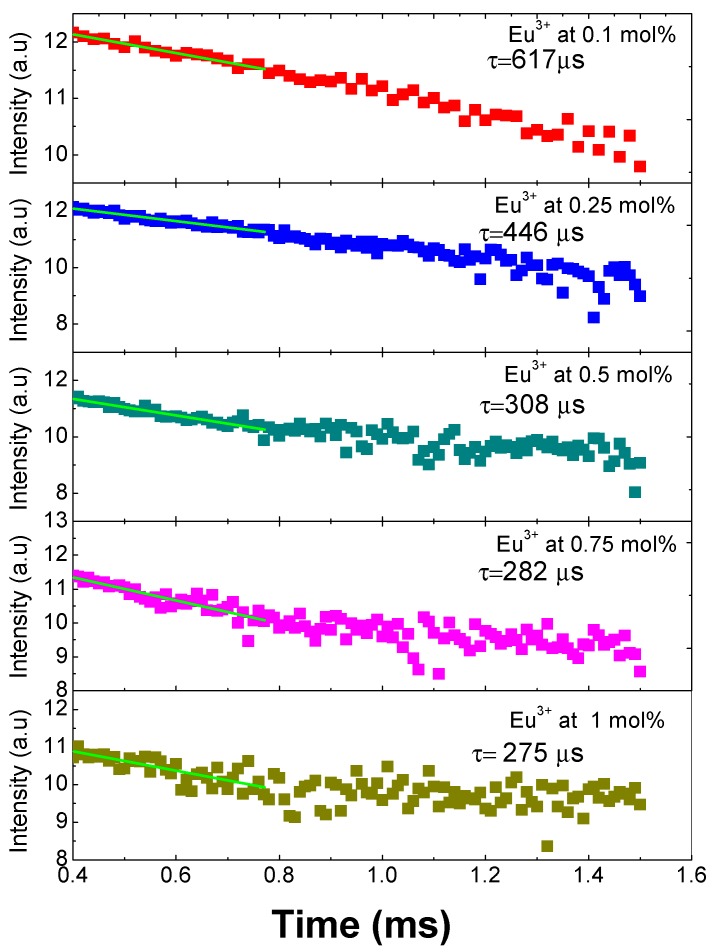
Decay curves and lifetimes of SiO_2_-PMMA:Eu^3+^ samples with concentration 0.1 mol % (red points), 0.25 mol % (blue points), 0.5 mol % (cyan points), 0.75 mol % (pink points), and 1 mol % (dark yellow points).

The decay time curves for all the doped samples first were fitted to a single exponential function with the following expression *I* = *I*_0_ *exp*(*−t/τ*), which indicates that the Eu^3+^ ions are located in similar sites suffering the same crystal field [4]. Subsequently, applying the natural logarithm function to these results, we obtain what is presented graphically in Figure 6, where it can be observed that the ^5^D_0_ lifetime shortens with the increase of the concentration of the Eu^3+^ ions due to the cross-relaxation process among Eu^3+^ ions. The lifetimes obtained are 275 ± 10 µs (1 mol %), 285 ± 6 µs (0.75 mol %), 308 ± 5 µs (0.5 mol %), 446 ± 20 µs (0.25 mol %), and 617 ± 30 µs (0.1 mol %). The cross-relaxation process leads to fluorescence quenching (i.e., to the decrease of fluorescence intensity when the rare earth concentration is increased). One has to consider, in fact, that, with the increasing concentration of rare earth ions, the ion spacing decreases and may be small enough to allow them to interact and transfer energy [18]. 

Another aspect that contributes to the lifetime shortening is the existence of hydroxyl groups in all the samples due to the fact that they were made at room temperature and were not submitted to a thermal treatment. Those groups have been observed to reduce the lifetime emission in silica matrix doped with Eu^3+^ when it has not been submitted to a thermal treatment [19]. For this matrix, the reported lifetimes are between 230 and 776 μs and are temperature dependent. To sum up, because the ionic radius of Eu^3+^ (1.07 Å) is larger than that of Si^4+^ (0.4 Å), the amount of Eu^3+^ in the matrix is restricted, and it can give place to the formation of clusters of the ion in the matrix that contributes to the cross relaxations [20]. 

One can also note that in a work by Basu and Vasantharajan [6], the decay lifetimes of Eu^3+^ in three different host matrices of polystyrene, polymethylmethacrylate, and polyurethane were compared. They observed that the luminescence intensity and the lifetime were strongly dependent on the matrix type, the temperature, and the amount of oxygen present. In particular, they found that the fastest time decay occurred in the PMMA matrixes, and it is around 364.2 μs.

## 4. Conclusions

Hybrid materials with optical properties are attracting more and more attention, especially in the application areas of sensing, imaging, and energy. Rare-earth-doped hybrid materials, in particular, may be optimized by playing with the interactions between the organic and the inorganic parts. Here, using the sol-gel method, a series of PMMA-silica hybrid materials, namely one undoped and five Eu^3+^-doped samples, were synthetized and optically characterized. The transparency of the undoped sample as a result of its low absorption in the visible range guarantees that it can have applications in photonic devices, for example, in the fabrication of organic light emitting diodes (OLEDs), microstructured polymer optical fibres (MPOFs), or polymer light emitting diodes (PLEDs). Also, the undoped and doped samples, as a hybrid coating, can work as a chemical detector through the change in the spectral signature, for example, to detect solvents, humidity, or proteins.

An energy transfer from the matrix of SiO_2_-PMMA to the Eu^3+^, doped at 1 mol %, could be observed in the emission spectra of the sample. This energy was absorbed at 393 nm and used to move the electrons from the ^7^F_0_ to the ^5^L_6_ level, which then relaxed using nonradiative transitions to the ^5^D_0_ level and finally emitted at 616 nm. The emission spectra were dopant concentration dependent. No quenching was found for the emission at the dopant concentrations used, but a decrease in the decay lifetime associated with the increment of dopant concentration was observable. It was attributed to a drop in the distance between the ions of Eu^3+^ and associated with the increment of cross relaxations and non-radiative transitions. The possibility of playing with the organic-inorganic structure (even at a nanoscale) on one side and with the rare earth concentration on another side, opens good prospects for the development of efficient luminescent devices in these hybrid materials.

## Figures and Tables

**Table 1:** Dopant concentration (mol %) vs direct gap energy (eV) for the six samples.

Concentration of Eu^3+^ (mol %)	Direct Gap (eV)
0	3.97 ± 0.20
0.1	4.01 ± 0.10
0.25	3.97 ± 0.12
0.5	3.98 ± 0.22
0.75	3.96 ± 0.12
1	4.01 ± 0.22
